# TP53 Loss Fuels mTORC1 Activation and Autophagy Suppression to Drive Immune-Cold Colorectal Cancer

**DOI:** 10.14740/wjon2695

**Published:** 2026-03-05

**Authors:** Eunseuk Lee, Dana Al-Assi, Randy Rivera-Rueda, Sharon Susan Paul, Aksa Joy

**Affiliations:** a Rutgers Health Community Medical Center, Toms River, NJ, USA; b The State University of New Jersey Rutgers, New Brunswick, NJ, USA

**Keywords:** TP53, mTORC1, Autophagy, Colorectal cancer, Immunometabolism, Proteomics, Immune evasion, Regulatory T cells

## Abstract

**Background:**

Microsatellite-stable colorectal cancer (MSS CRC) is typically resistant to immune checkpoint blockade and remains an “immune-cold” disease. Wild-type p53 is known to restrain mTOR signaling and support autophagy, yet how TP53 loss integrates metabolic rewiring with immune suppression in MSS CRC—especially with protein- and phosphosite-level validation—remains incompletely defined. We tested whether p53 deficiency is associated with coordinated mTORC1 activation, autophagy attenuation, and immune-cold remodeling across multi-omics datasets.

**Methods:**

We analyzed GSE146009 (paired tumor-normal RNA-seq pairs), TCGA-COAD/READ (n = 647, mutation annotated), GSE108989 (11,138 tumor-infiltrating T cells), and CPTAC colon proteome/phosphoproteome cohorts. Pathway activities were quantified by single-sample gene set enrichment analysis (ssGSEA) and Seurat module scoring. Group differences were tested by Kruskal–Wallis and Wilcoxon analyses with false-discovery-rate correction. CPTAC phosphosite-to-protein ratios were compared between TP53-mutant or p53-low versus wild-type or p53-high tumors.

**Results:**

Across bulk transcriptomes, TP53-mutant tumors showed higher mTORC1 signaling, lower autophagy scores, higher FOXP3, and reduced CD8A/FOXP3 ratios consistent with a regulatory T-cell-skewed immune-cold state. Single-cell analysis confirmed subset-specific immunometabolic programs, highlighting metabolically active regulatory/exhausted states with relatively blunted inflammatory output. Proteomic/phosphoproteomic data supported post-translational mTOR pathway activation in p53-deficient tumors, including increased phosphorylation of canonical mTOR substrates (EIF4EBP2 S65; RPS6KB1 T421/S424) and reduced phosphorylation at inhibitory RPTOR sites (S705/T725/S726), consistent with sustained mTORC1 activity.

**Conclusions:**

In MSS CRC, p53 deficiency is consistently associated with immunometabolic remodeling marked by persistent mTOR pathway activation, relative autophagy attenuation, and FOXP3-dominant immune-cold features across transcriptomic, single-cell, and proteomic layers. These findings add protein- and phosphosite-level evidence linking TP53 loss to an immune-suppressive metabolic state and support biomarker-guided evaluation of mTOR kinase (TORC1/2) inhibition, which more fully suppresses 4E-BP/S6K phosphorylation, combined with PD-1/PD-L1 (± CTLA-4) blockade in TP53-deficient MSS CRC.

## Introduction

Colorectal cancer (CRC) remains one of the leading causes of cancer-related deaths worldwide, and most cases are microsatellite-stable (MSS) rather than microsatellite instability-high (MSI-H) [1–5]. In contrast to MSI-H tumors, which frequently respond to immune checkpoint inhibitors (ICIs), MSS CRC is largely refractory to immunotherapy. This resistance reflects an immunologically “cold” tumor microenvironment (TME) characterized by limited cytotoxic T-cell infiltration, low neoantigen-driven immunogenicity, and dominant immunosuppressive signaling networks. Understanding the molecular mechanisms that maintain this immune-cold state remains a major unmet need in CRC [4–8].

Among the genetic alterations shaping the MSS phenotype, loss of TP53 function is one of the most common and biologically consequential events, occurring in nearly half of all CRCs [9–14]. Beyond its canonical role in genomic surveillance, wild-type p53 serves as a central regulator of cellular metabolism by restraining growth-promoting pathways and promoting energy homeostasis. A key downstream consequence of TP53 loss is dysregulation of the PI3K–AKT–mTOR axis, resulting in sustained anabolic signaling, enhanced proliferation, and therapeutic resistance [15–22]. Under physiologic conditions, p53 suppresses mTORC1 activity through AMPK- and ULK-dependent mechanisms and promotes autophagy to buffer metabolic stress [23]. When p53 function is lost, this regulatory balance is disrupted, allowing unchecked mTORC1 activation and suppression of autophagic programs [3, 24–31].

Autophagy impairment itself has important consequences for both progression and immune regulation. In CRC, reduced autophagic flux has been linked to diminished antigen presentation, impaired cytotoxic T-cell priming, and resistance to immunotherapy [32–35]. Expression of autophagy-related genes such as ATG16L1 has been associated with poor response to PD-1/PD-L1 blockade in MSS disease, underscoring the clinical relevance of this pathway in immune-refractory CRC. At the same time, hyperactive mTORC1 signaling reshapes the TME by increasing nutrient competition and metabolic stress, thereby favoring immunosuppressive cell populations and weakening effector T-cell function [29, 30, 36–45].

Collectively, these findings suggest that TP53 loss may couple metabolic hyperactivation with immune suppression in MSS CRC through coordinated dysregulation of the mTOR1-autophagy axis. While prior studies have independently linked p53 loss to mTOR activation and mTORC1 signaling to immunosuppressive TME, how these processes integrate across molecular layers in MSS CRC remains incompletely defined [20, 22, 24, 46–49]. In particular, protein- and phosphosite-level validation of TP53-associated mTORC1 activity in relation to immune contexture has been limited. We therefore aimed to determine whether TP53 loss is associated with coordinated immunometabolic reprogramming through the mTORC1–autophagy axis in MSS CRC. Using integrated analyses of bulk transcriptomics, single-cell RNA sequencing of tumor-infiltrating T cells, and CPTAC proteomic and phosphoproteomic datasets, we tested the hypothesis that p53 deficiency is linked to persistent mTORC1 activation, relative autophagy suppression, and development of a FOXP3-dominant, immune-cold TME. By combining multiple omics layers across independent cohorts, this study seeks to provide mechanistic and post-translational context to the role of TP53 loss in shaping immune resistance in MSS CRC [50–62].

## Materials and Methods

### Study design and data sources

This study was designed as a multi-cohort, multi-omics analysis of how TP53 dysfunction is associated with metabolic and immune pathways remodeling in CRC, with a focus on MSS disease. All analyses were conducted using publicly available human datasets: 1) GSE146009 (discovery cohort): Bulk RNA-seq from 24 paired colorectal tumor and adjacent normal tissues generated on the Illumina HiSeq platform. 2) TCGA-COAD/READ (validation cohort): RNA-seq and mutation annotation data from 647 primary colorectal adenocarcinomas obtained through the Genomic Data Commons (GDC) portal. Tumors were categorized as *TP53-wild type*, *missense*, or *null (loss-of-function)* based on variant classification. 3) GSE108989 (single-cell cohort): Single-cell RNA sequencing of 11,138 tumor-infiltrating T cells isolated from 12 CRC patients. 4) CPTAC colon proteome/phosphoproteome datasets (PDC000111, PDC000116-117): Quantitative proteomic and phosphosite profiles generated by the Clinical Proteomic Tumor Analysis Consortium.

### Bulk RNA-seq processing and pathway analysis

For bulk transcriptomic data (GSE146009 and TCGA-COAD/READ), normalized expression values were analyzed as log_2_(FPKM + 1) or log_2_(counts-per-million + 1), depending on dataset availability. Pathway activities were quantified using single-sample gene set enrichment analysis (ssGSEA) implemented in the GSVA R package. We prioritized MSigDM Hallmark pathways for their robustness and reduced redundancy across platforms, complemented by Reactome pathways to capture mechanistic detail for autophagy-related processes. Pathways of interest included *mTORC1* signaling, *p53* signaling, *autophagy*, *TNF-α/NF-κB* signaling, and *interferon-γ* response.

To estimate immune effector-regulatory balance, a CD8A/FOXP3 ratio was calculated for each sample using the formula:

$$\frac{\text{CD8A}+\text{0.1}}{\text{FOXP3}+\text{0.1}}$$

This ratio was used as a continuous index of cytotoxic versus regulatory T-cell predominance.

### Single-cell RNA-seq analysis

The GSE108989 dataset was processed using Seurat v4.4. Cells with fewer than 200 detected genes or more than 15% mitochondrial transcripts were excluded. Expression data were log-normalized and scaled; variable genes were identified with *FindVariableFeatures()*. Module scores for *mTORC1*, *autophagy*, and *IFNG* gene sets were calculated using *AddModuleScore()* and z-scaled to enable comparison across T-cell subsets. T-cell populations were annotated based on established marker expression: CD8_eff (CD8A^+^ GZMB^+^), CD8_exh (CD8A^+^ PDCD1^+^ or HAVCR2^+^), TH1_CXCL13 (CXCL13^+^ BHLHE40^+^), Treg (FOXP3^+^ IL2RA^+^), and Other_T (unclassified or naive cells). Subset labels were stored in the Seurat metadata for downstream analyses.

### Proteomic and phosphoproteomic analyses

CPTAC proteomic and phosphoproteomic data were downloaded from the Proteomic Data Commons (PDC). Raw peptide intensities were log_2_-transformed and normalized to total protein abundance. TCGA barcodes were used to align samples across genomic and proteomic datasets when applicable.

Tumors were stratified either by TP53 mutation status (mutant vs. wild type) or by TP53 protein abundance, dichotomized at the cohort-wide median into *p53*-low and *p53*-high groups. Median-based stratification was chosen to avoid arbitrary thresholds, preserve statistical power, and ensure reproducibility across cohorts lacking established clinical cutpoints for p53 protein expression. Phosphosites-level differences were assessed using the Wilcoxon rank-sum test, with P-values adjusted using the Benjamini-Hochberg false-discovery rate (FDR). Phosphosites were considered biologically relevant based on consistent directionality, effect size (log_2_fold-change > 0.5), and nominal statistical significance.

### Statistical and correlation analyses

All analyses were performed in R version 4.5.1 using tidyverse, ggpubr, ComplexHeatmap, and igraph.

#### Correlations analyses

Pearson’s correlation was used for linear relationships with Spearman’s rank correlation applied for robustness where appropriate. Fisher’s r-to-z transformation was used to compare correlation coefficients across *TP53* classes or T-cell subsets.

#### Group comparisons

Differences across multiple groups were evaluated using the Kruskal–Wallis test, followed by pairwise Wilcoxon rank-sum tests for post-hoc comparisons.

#### Confounders assessment

Exploratory multivariable linear models including age, sex, and tumor stage were used to ensure observed associations were not driven by clinical covariates.

#### Significance threshold

Two-sided FDR-adjusted P < 0.05 was considered statistically significant.

### Principal component and derived index analyses

Principal component analysis (PCA) was performed on scaled pathway scores for *mTORC1*, *autophagy*, *IFNG* to identify major axes of immunometabolic variation. The first component (PC1) captured overall metabolic intensity, while the second component (PC2) reflected immune–metabolic divergence. A Metabolic-Immune Index, defined as (*mTORC1–autophagy*), was plotted against *IFNG* activity to visualize metabolic-immune polarization across tumor-infiltrating T-cell subsets.

Figures were created in BioRender and RStudio using *ggplot2 v3.5.0* for statistical plots, *ComplexHeatmap* for correlation matrices, and *patchwork* for multi-panel layouts.

### Institutional Review Board (IRB) approval

This study analyzed de-identified, publicly accessible human data from TCGA, CPTAC, and GEO repositories and was therefore exempt from institutional review board approval.

### Ethical compliance with human study

This study was conducted in compliance with the ethical standards of the Declaration of Helsinki and all relevant national and institutional guidelines for research using human data.

## Results

### Transcriptomic profiling reveals mTORC1–autophagy antagonism in colorectal tumors

**Figure 1:**
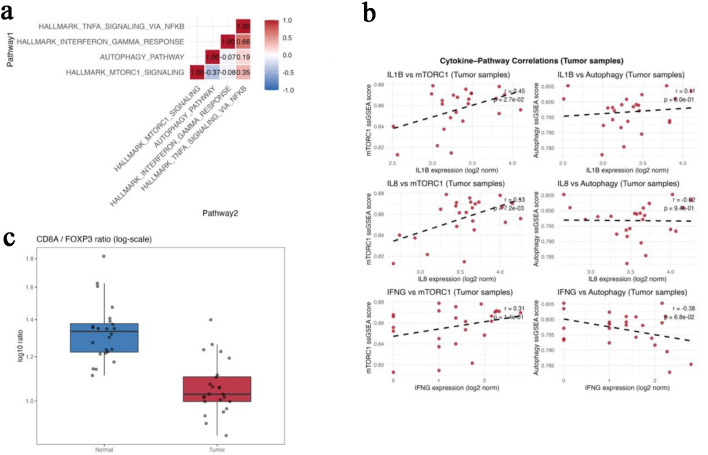
Transcriptomic profiling reveals mTORC1-autophagy antagonism in colorectal tumors (GSE146009). (a) Correlation matrix showing pairwise relationships among *mTORC1* signaling, *autophagy*, *interferon-γ* response, *TNF-α/NF-κB* signaling, and *p53* pathway *s*cores in paired tumor and normal tissues. (b) Scatterplots showing positive correlations between *mTORC1* and *IL1B*/*IL8* expression and an inverse correlation between autophagy and *IFNG*. (c) Boxplot comparing the CD8A/FOXP3 ratio between paired normal and tumor tissues. Gene-level expression differences for FOXP3, CTLA4, CD274 (PD-L1), and CD8A are summarized in Table 1.

Analysis of the discovery cohort (GSE146009) demonstrated consistent transcriptional differences between paired colorectal tumors and adjacent normal tissues. Correlation analysis across five hallmark pathways—*mTORC1* signaling, *autophagy*, *interferon-γ* response, *TNF-α/NF-κB* signaling, and *p53* signaling—showed that *mTORC1* activity was positively correlated with *TNF-α/NF-κB* (r = 0.35) and inversely correlated with *autophagy* (r = −0.37) (Fig. 1a), suggesting reciprocal regulation between anabolic and catabolic programs in tumor tissue.

At the cytokine level, *mTORC1* pathway scores correlated positively with IL1B (r = 0.45, P = 0.027) and IL8 (r = 0.53, P = 0.007), whereas *autophagy* scores showed inverse relationship with IFNG expression (r = −0.38, P = 0.068) (Fig. 1b). These associations are consistent with an inflammatory but immunoregulatory transcriptional state.

Gene-level comparisons demonstrated higher expression of FOXP3, CTLA4, and CD274 (PD-L1) in tumor tissue compared with matched normal samples, while CD8A expression remained unchanged (Table 1). Accordingly, the CD8A/FOXP3 ratio was significantly lower in tumors than in normal tissue (1.06 ± 0.12 vs. 1.34 ± 0.17; Kruskal-Wallis P < 0.01) (Fig. 1c), indicating a relative shift toward regulatory immune features.

**Table 1:** Differential Expression of Immune-Related Genes Between Normal and Tumor Tissues (GSE146009)

Gene	Normal (mean ± SD)	Tumor (mean ± SD)	Fold-change (log_2_)	P-value
*FOXP3*	2.24 ± 0.26	2.76 ± 0.31	+0.52	0.003
*CTLA4*	2.37 ± 0.30	2.69 ± 0.33	+0.32	0.018
*CD274 (PD-L1)*	2.65 ± 0.29	2.89 ± 0.34	+0.24	0.022
*CD8A*	2.98 ± 0.41	2.92 ± 0.39	−0.06	0.712

### TP53 mutation class is associated with differential metabolic-immune pathway coupling

**Figure 2:**
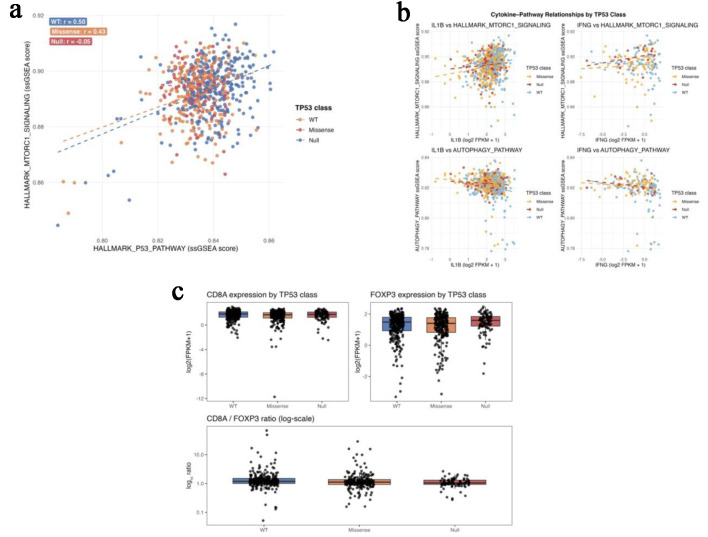
TP53 mutation class predicts metabolic and immune pathway decoupling in TCGA-COAD/READ. (a) Scatterplots of *P53*-pathway and *mTORC1*-signaling ssGSEA scores across TP53-wild-type, missense, and null tumors. (b) Correlation plots showing negative *autophagy-cytokine* coupling in wild-type tumors and positive *mTORC1-IFNG* coupling in TP53-null tumors. (c) Boxplots of *FOXP3* expression and CD8A/FOXP3 ratios across TP53 functional classes.

In the TCGA-COAD/READ cohort (n = 647), tumors were classified as TP53-wild type (n = 258), missense (n = 287), and null (loss-of-function) (n = 102). Across these groups, coupling between *p53* pathway activity and *mTORC1* signaling progressively weakened. Strong positive correlation was observed in TP53-wild type tumors (r = 0.50; 95% CI 0.41–0.57), which declined in missense tumors (r = 0.43; 95% CI 0.35–0.50) and was lost in TP53-null tumors (r = −0.05; 95% CI −0.25 to 0.15) (Fig. 2a).

Within TP53-wild type tumors, autophagy scores showed modest inverse correlations with *IL1B* (r = −0.20) and *IFNG* (r = −0.20), whereas *mTORC1* and *IFNG* were not significantly associated. In contrast, TP53-null tumors demonstrated a positive correlation between *mTORC1* and *IFNG* expression (r = 0.28, P = 0.0045), accompanied by loss of autophagy-cytokine coupling (Fig. 2b).

Expression of *FOXP3* increased stepwise from wild-type to missense to null TP53 classes (Kruskal–Wallis P = 0.005), while *CD8A* expression remained stable across groups. As a result, the *CD8A/FOXP3* ratio declined progressively with increasing TP53 dysfunction (wild type > missense > null; P = 4.7 × 10^−5^) (Fig. 2c; Table 2), indicating a TP53-associated shift toward regulatory immune dominance.

**Table 2:** TP53 Mutation Class and Immune Gene Expression Summary (TCGA-COAD/READ)

TP53 class	n	FOXP3 (mean ± SD)	CD8A/FOXP3 ratio	P (Kruskal–Wallis)
Wild type	258	2.42 ± 0.36	1.28 ± 0.14	–
Missense	287	2.59 ± 0.39	1.11 ± 0.16	0.005
Null	102	2.71 ± 0.43	0.96 ± 0.15	4.7 × 10^−5^

### Single-cell RNA sequencing confirms subset-specific immunometabolic heterogeneity

**Figure 3:**
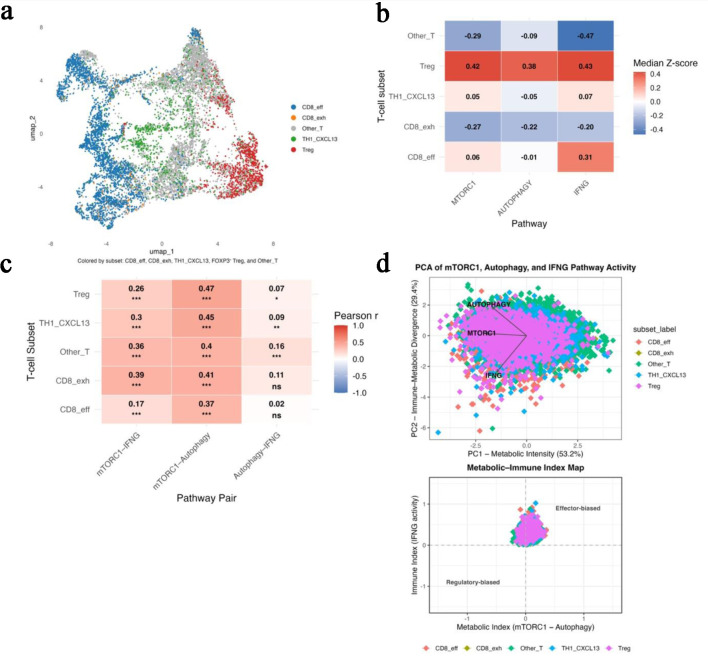
Single-cell RNA-seq analysis confirms subset-specific metabolic heterogeneity (GSE108989). (a) UMAP projection identifying five major T-cell subsets: CD8_eff, CD8_exh, TH1_CXCL13, Treg, and Other_T. (b) Heatmap of median z-scores for *mTORC1*, *autophagy*, and *IFNG* pathway activity across T-cell subsets, illustrating metabolic polarization. (c) Heatmap showing pairwise correlation strengths among *mTORC1-autophagy*, *mTORC1*-*IFNG*, and *autophagy-IFNG* within each subset. (d) Principal-component analysis illustrating PC1 (metabolic intensity) and PC2 (immune-metabolic divergence) with the derived Metabolic-Immune Index map separating effector and regulatory T-cell states.

After quality control of the GSE108989 dataset, 11,138 tumor-infiltrating T cells were retained for analysis. Uniform manifold approximation and projection (UMAP) projection identified five major T-cell populations: CD8_eff, CD8_exh, TH1_CXCL13, Treg, and Other_T (Fig. 3a).

Analysis of z-scored module scores demonstrated marked immunometabolic heterogeneity across subsets (Fig. 3b). *Tregs* exhibited the highest combined activity of *mTORC1*, *autophagy*, and *IFN-γ-*response pathways, consistent with a metabolically active regulatory phenotype. *CD8_eff* cells showed moderate *mTORC1* and *IFN-γ* activity with near-basal autophagy, whereas *CD8_exh* cells displayed uniformly low pathway activity, consistent with metabolic exhaustion. *TH1_CXCL13* cells demonstrated intermediate activity, and *Other_T* cells remained largely quiescent.

Pairwise correlation analysis within each subset showed that *mTORC1–autophagy* coupling was consistently the strongest relationship (mean r ≈ 0.42 ± 0.04), followed by weaker associations between *mTORC1* and *IFN-γ* (r ≈ 0.30 ± 0.09) and minimal coupling between *autophagy* and IFN-γ (r ≈ 0.09 ± 0.05) (Fig. 3c). Principal component analysis integrating all three pathways revealed two dominant r axes of variation, separating metabolically intense states from immune-dominant effector states (Fig. 3d, Table 3). Together, these findings indicate that tumor-infiltrating T cells occupy distinct immunometabolic states that parallel the bulk-tumor associations observed with TP53 dysfunction.

**Table 3:** Summary of Principal Component and Metabolic-Immune Index Analyses Across T-Cell Subsets (Fig. 3d)

Subset	PC1 (metabolic intensity)	PC2 (immune-metabolic divergence)	Metabolic index (mTORC1–autophagy)	IFN-γ index
Treg	−0.72	−0.14	0.06 ± 0.07	0.30 ± 0.10
CD8_eff	−0.28	−0.31	0.05 ± 0.08	0.30 ± 0.09
TH1_CXCL13	−0.11	−0.12	0.06 ± 0.07	0.27 ± 0.10
CD8_exh	+0.34	+0.04	0.04 ± 0.07	0.23 ± 0.07
Other_T	+0.37	+0.26	0.03 ± 0.08	0.22 ± 0.09

### Proteomic and phosphoproteomic integration validate post-translational mTORC1 activation under p53 deficiency

**Figure 4:**
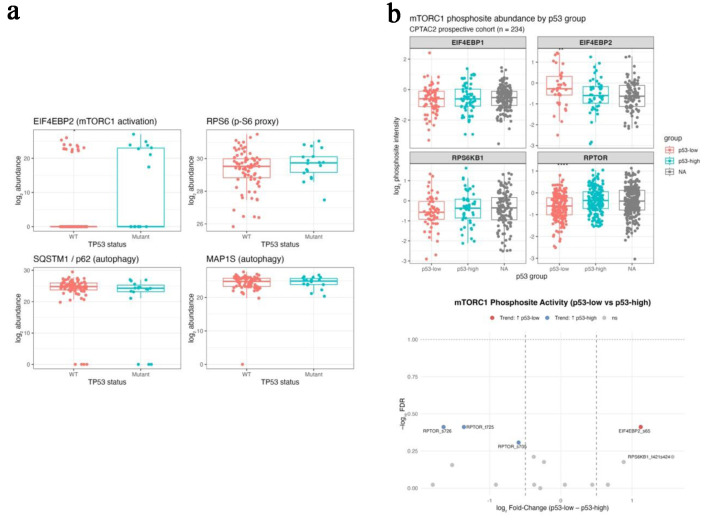
Proteomic and phosphoproteomic integration validates post-translational mTORC1 activation under p53 deficiency (CPTAC colon cohorts). (a) Boxplots comparing protein abundance of EIF4EBP2, RPS6, SQSTM1 (p62), and MAP1S between TP53-mutant and wild-type tumors in the CPTAC-TCGA matched proteome. (b) Bar (upper) and volcano (lower) plots from the CPTAC2 phosphoproteome showing increased phosphorylation of EIF4EBP2 S65 and RPS6KB1 T421/S424 and decreased phosphorylation of inhibitory RPTOR sites (S705, T725, S726) in p53-low tumors.

To determine whether transcriptomic associations extended to the protein level, CPTAC CRC proteomic and phosphoproteomic datasets were analyzed. In the CPTAC–TCGA matched proteome (PDC000111, n = 95), TP-mutant tumors showed increased abundance of EIF4EBP2 (log_2_ fold-change = +5.98; FDR = 0.07) and modest increase in RPS6 (log_2_ fold-change = +0.34; FDR = 0.42), alongside reduced SQSTM1/p62 (log_2_ fold-change = 23.07; FDR = 0.30) with minimal change in MAP1S (Fig. 4a; Table 4). Although not all differences reached statistical significance after FDR correction, the directionality of changes was consistent with increased mTOR pathway activity and reduced autophagy-related protein accumulation.

**Table 4:** Protein-Level Differences by TP53 Mutation Status (CPTAC–TCGA PDC000111)

Protein	Mean (WT)	Mean (mutant)	Log_2_ FC (Mut – WT)	Direction of change
EIF4EBP2	4.25	10.23	+5.98	↑ in TP53-mutant
RPS6	29.31	29.65	+0.34	↑ in TP53-mutant
SQSTM1 (p62)	23.58	20.51	−3.07	↓ in TP53-mutant
MAP1S	24.20	24.41	+0.20	≈ No change

Data from the CPTAC-TCGA matched colorectal proteome (PDC000111). Log_2_ fold-change represents mean abundance difference between TP53-mutant and wild-type tumors.

In the CPTAC2 phosphoproteomic cohort (PDC000116-000117, n = 234), tumors with low p53 protein abundance demonstrated increased phosphorylation of canonical mTOR output sites, including EIF4EBP2 S65 and RPS6KB1 T421/S424 (log_2_ fold-change = +1.1 to +1.6). In contrast, phosphorylation of RPTOR sites (S705/T725/S726) was reduced in p53-low tumors and enriched in p53-high tumors (log_2_ fold-change = −0.6 to −1.6) (Fig. 4b; Table 5). These phosphosite patterns are consistent with sustained mTOR pathway output in the setting of reduced p53 protein levels.

**Table 5:** Differential Phosphosite Abundance by p53 Protein Level (CPTAC2 PDC000117–000116)

Gene	Phosphosite	log_2_ FC (p53-low – p53-high)	Direction of change
*EIF4EBP2*	S65	+1.12	↑ in p53-low
*RPS6KB1*	T421/S424	+1.57	↑ in p53-low
*RPTOR*	S705	−0.60	↑ in p53-high
*RPTOR*	T725	−1.36	↑ in p53-high
*RPTOR*	S726	−1.65	↑ in p53-high
*RPTOR*	S719	−0.38	↑ in p53-high

Data from the prospective CPTAC2 colon and rectal phosphoproteome (PDC000116-000117, n = 234). Positive log_2_FC indicates higher phosphorylation in p53-low tumors; negative values indicate higher phosphorylation in p53-high tumors.

### Integrated multi-omics summary

Integration of bulk transcriptomic, single-cell, and proteomic analyses revealed a consistent association between TP53 dysfunction and coordinated immunometabolic remodeling in CRC. Across independent cohorts, TP-53 mutant or p53-low tumors were characterized by increased mTOR pathway activity, relative attenuation of autophagy-related programs, and immune features indicative of a regulatory-dominant immune-cold TME.

At the transcriptomic level, TP53-altered tumors showed higher mTORC1 signaling scores and lower CD8A/FOXP3 ratios, reflecting a shift toward FOXP3-associated immunoregulation. Single-cell analysis of tumor-infiltrating T cells demonstrated parallel immunometabolic states, with regulatory and exhausted T-cell subsets occupying metabolically active but functionally constrained niches. Proteomic and phosphoproteomic data provided post-translational context to these associations, showing increased phosphorylation of canonical mTOR output sites and reduced phosphorylation of inhibitory regulatory sites in p53-deficient tumors.

Taken together, these convergent observations across molecular layers support a unified model in which TP53 loss is associated with sustained mTOR pathway output, altered autophagy-related signaling, and immune-cold remodeling in CRC. This integrated view provides a mechanistic framework for interpreting TP53-associated immune resistance in MSS disease and informs the therapeutic rationale discussed below.

## Discussion

This study provides an integrated multi-omics view of how TP53 dysfunction is associated with immunometabolic remodeling in MSS CRC. By combining bulk transcriptomic profiling, single-cell analysis of tumor-infiltrating T cells, and CPTAC proteomic and phosphoproteomic data, we identified a consistent pattern characterized by increased mTOR pathway activity, relative attenuation of autophagy-related programs, and immune-cold features marked by FOXP3 enrichment and reduced CD8A/FOXP3 ratios. Rather than relying on a single molecular layer, our approach emphasizes convergent evidence across independent datasets and platforms, strengthening the biological plausibility of these associations [63–71].

At the transcriptomic level, TP53-mutant tumors exhibited higher mTORC1 signaling scores and lower autophagy activity compared with TP53-wild type tumors. These metabolic alterations were accompanied by increased expression of immunoregulatory genes, including FOXP3, CTLA4, and CD274, and by a progressive reduction in the CD8A/FOXP3 ratio. These observations align with the established role of wild-type p53 in restraining mTORC1 activity and promoting autophagy under conditions of metabolic stress. Loss of this regulatory function is known to permit sustained anabolic signaling, and our data suggest that, in MSS CRC, this metabolic shift is accompanied by immune remodeling toward a more suppressive TME [1, 22, 30, 66–70, 72–74].

Single-cell RNA sequencing extended these associations to the immune compartment. Tumor-infiltrating T cells displayed distinct immunometabolic programs linked to functional state. Regulatory T cells (Tregs) and exhausted CD8^+^ T cells occupied metabolically active yet cytokine-constrained states, whereas cytotoxic CD8^+^ effector and TH1-like T cells demonstrated stronger IFNG signaling with more balanced metabolic activity. Although TP53 mutation status was not available at the single-cell level, these subset-specific patterns parallel the bulk-tumor associations observed in TP53-deficient cancers and are consistent with prior work linking mTORC1 signaling to T-cell exhaustion and Treg expansion. Together, these findings suggest that tumor-intrinsic metabolic rewiring associated with TP53 loss may indirectly shape immune-cell function within the TME [30, 31, 75–81].

A key strength of this study is the incorporation of proteomic and phosphoproteomic data, which provide post-translational context to the transcriptomic and cellular findings. In CPTAC cohorts, p53-deficient or p53-low tumors demonstrated increased phosphorylation of canonical mTOR pathway output nodes, including EIF4EBP2 and RPS6KB1, alongside reduced phosphorylation of inhibitory RPTOR sites targeted by AMPK and ULK1 [66–70]. Although not all phosphosite differences reached stringent false-discovery thresholds, the consistent directionality across multiple regulatory nodes supports sustained mTOR pathway output in the setting of reduced p53 activity. This protein-level validation adds mechanistic depth beyond expression-based analyses and addresses a major limitation of prior single-omics studies.

From a clinical perspective, these findings are particularly relevant in MSS CRC, a disease subtype that remains largely resistant to immune checkpoint blockade despite frequent immune-cell infiltration. Current evidence suggests that immune resistance in MSS CRC is not driven solely by low neoantigen burden but is also shaped by metabolic constraints within the TME [4–6, 8, 44, 82]. Our data support this concept by linking TP53-associated mTOR pathway activation to immune-cold features providing a framework for understanding why immune checkpoint inhibitors have limited efficacy in this setting.

**Figure 5:**
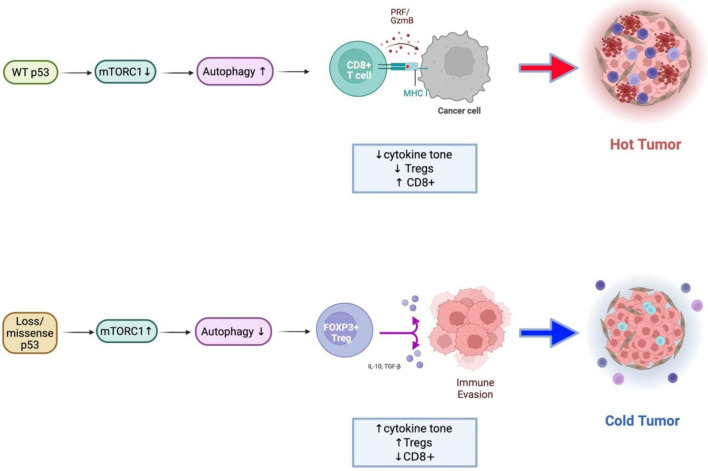
Conceptual model of the TP53–mTORC1–autophagy–immune axis in colorectal cancer. Schematic illustration summarizing the proposed mechanism derived from integrated transcriptomic, single-cell, and proteomic analyses. Upper: In wild-type p53 tumors, p53 restrains mTORC1 activity through AMPK/ULK1-mediated phosphorylation of RPTOR and promotes autophagy, maintaining metabolic balance and supporting effective antitumor immune function. Lower: In TP53-mutant or null tumors, loss of p53 control leads to constitutive mTORC1 activation and suppression of autophagy. These changes drive increased cytokine signaling (IL1B, IFNG), metabolic stress adaptation, and enrichment of FOXP3^+^ regulatory T cells, resulting in an immune-cold, immunosuppressive tumor microenvironment.

Importantly, this study builds on prior work describing p53–mTOR crosstalk and mTOR-driven immunosuppression by integrating genomic, transcriptomic, single-cell, and post-translational proteomic layers within MSS CRC specifically. While earlier studies have demonstrated that TP53 loss can activate mTOR signaling and that high mTORC1 activity correlates with immunosuppressive TME, our findings extend this literature by providing phosphosite-level evidence linking TP53 dysfunction to sustained mTOR pathway output in association with FOXP3-dominant immune remodeling [22, 24, 30, 46, 83]. This integrative approach represents a key contribution beyond mutation-centric or transcriptomics-only analysis (Fig. 5).

These observations have potential implications for therapeutic strategy. The phosphoproteomic signatures observed in p53-deficient tumors implicate downstream mTOR output nodes, suggesting that mTOR kinase inhibition (targeting both TORC1 and TORC2) may be more effective than selective TORC1 inhibition alone. In this context, combining mTOR pathway inhibition with immune checkpoint blockade may help restore metabolic balance and improve antitumor immune function in TP53-mutant MSS CRC, as supported by emerging preclinical and early clinical data [63, 64, 84–87]. TP53 status, together with markers of mTOR pathway activity and immune balance, may therefore serve as useful biomarkers for patient stratification in future trials.

Several limitations should be acknowledged. This study is retrospective and hypothesis-generating, and the associations described do not establish causality. Functional validation in experimental models will be required to directly test whether TP53 loss drives mTOR-dependent autophagy suppression and immune remodeling. In addition, other components of the TME, including stromal, myeloid, and microbiome-related factors, may further influence immunometabolic states beyond TP53 status alone [4, 8, 88]. Nonetheless, the consistency of findings across multiple omics layers and independent cohorts supports the robustness of the observed associations.

In summary, this study identifies a reproducible, multi-omics signature linking TP53 dysfunction with sustained mTOR pathway activity, altered autophagy-related signaling, and immune-cold remodeling in CRC. By integrating transcriptomic, single-cell, and proteomic data, these findings provide mechanistic context for immune resistance in MSS CRC and support rational, biomarker-informed strategies targeting the TP53–mTOR–autophagy axis to overcome therapeutic resistance.

## Data Availability

Bulk and single-cell transcriptomic datasets are available through the NCBI Gene Expression Omnibus (GSE146009, GSE108989). TCGA-COAD/READ datasets can be accessed through the Genomic Data Commons**,** and proteomic datasets (PDC000111, PDC000116-117) through the Proteomic Data Commons. Custom R scripts used for preprocessing, ssGSEA scoring, and single-cell module analysis are available from the corresponding author upon reasonable request.

## Abbreviations

AMPK: AMP-activated protein kinase
APC: antigen-presenting cell
ATR: ataxia telangiectasia and Rad3-related protein
ATG16L1: autophagy-related gene 16-like 1
BRAF: v-Raf murine sarcoma viral oncogene homolog B1
CD274 (PD-L1): programmed death-ligand 1
CD8A: cluster of differentiation 8 alpha
CRC: colorectal cancer
CPTAC: Clinical Proteomic Tumor Analysis Consortium
CTLA4: cytotoxic T-lymphocyte-associated protein 4
DRAM: damage-regulated autophagy modulator
EIF4EBP1/2: eukaryotic translation initiation factor 4E-binding protein 1/2
FDR: false discovery rate
FOXP3: forkhead box protein P3
FPKM: fragments per kilobase of transcript per million mapped reads
GDC: Genomic Data Commons
GEO: Gene Expression Omnibus
GOF: Gain of Function
GSVA: Gene Set Variation Analysis
IFNG: interferon gamma
ICI: immune checkpoint inhibitor
IL1B: interleukin 1 beta
IL2RA: interleukin 2 receptor alpha
IL8: interleukin 8
IRB: Institutional Review Board
KRAS: Kirsten rat sarcoma viral oncogene homolog
LC3B: microtubule-associated protein 1 light chain 3 beta
MAF: mutation annotation format
MAP1S: microtubule-associated protein 1S
mTORC1: mechanistic target of rapamycin complex 1
MSS: microsatellite stable
MSI-H: microsatellite instability-high
NCCN: National Comprehensive Cancer Network
NK: natural killer
OXPHOS: oxidative phosphorylation
p62 (SQSTM1): sequestosome 1
PCA: principal component analysis
PC1/PC2: principal component 1/principal component 2
PD-1: programmed cell death protein 1
PD-L1: programmed death-ligand 1
PDC: Proteomic Data Commons
PDTX: patient-derived tumor xenograft
PI3K: phosphatidylinositol 3-kinase
PKM2: pyruvate kinase M2 isoform
PLK1: Polo-like kinase 1
RPS6: ribosomal protein S6
RPTOR: regulatory associated protein of mTOR
RNA-seq: RNA sequencing
ROS: reactive oxygen species
ssGSEA: single-sample gene set enrichment analysis
TCA: tricarboxylic acid
TCGA: The Cancer Genome Atlas
TCR: T-cell receptor
Tfh: T follicular helper cell
TH1_CXCL13: T helper 1-like CXCL13-positive subset
TME: tumor microenvironment
TNFA: tumor necrosis factor alpha
TP53: tumor protein p53 gene
Treg: regulatory T cell
ULK1: Unc-51-like autophagy activating kinase 1
UMAP: uniform manifold approximation and projection
WT: wild type
